# Evaluation of Functional Outcomes After Arthroscopic All-Inside Meniscal Repair

**DOI:** 10.7759/cureus.104075

**Published:** 2026-02-22

**Authors:** H N Mahadeva, Raviraj Tantry, Amarnath Dasari, Aravind Devendrappa, Nishanth Reddy, Mohammed Shahid

**Affiliations:** 1 Orthopedics, Bangalore Medical College and Research Institute, Bangalore, IND; 2 Orthopedics, Vydehi Institute of Medical Sciences and Research Centre, Bangalore, IND

**Keywords:** all-inside meniscal repair, arthroscopy, ikdc score, knee function, lysholm score, meniscal tear

## Abstract

Introduction

Meniscal injuries are among the most common knee pathologies and play a significant role in the development of early osteoarthritis when inadequately treated. Preservation of the meniscus has therefore become a key principle in modern knee surgery. Arthroscopic all-inside meniscal repair has gained popularity due to reduced surgical morbidity, shorter operative time, and lower risk to neurovascular structures compared with traditional repair techniques. This study evaluates the functional outcomes and safety of the arthroscopic all-inside meniscal repair technique.

Methodology

This prospective study was conducted in the Department of Orthopedics at Vydehi Institute of Medical Sciences and Research Centre, Bangalore. A total of 38 patients diagnosed with meniscal injury were included. All patients underwent arthroscopic all-inside meniscal repair. Functional outcomes were assessed preoperatively and postoperatively at two weeks, three months, and six months using the Lysholm Knee Score, Tegner Activity Scale, International Knee Documentation Committee (IKDC) score, and Visual Analog Scale (VAS) for pain.

Results

There was a statistically significant improvement in all functional outcome measures following surgery. Mean Lysholm, IKDC, Tegner, and VAS scores showed progressive improvement at each follow-up interval compared with preoperative values. At the final six-month follow-up, the mean Lysholm score and Tegner Activity Scale improved from 64.4 ± 8.2 preoperatively to 94.05 ± 7.2, which was statistically significant (p < 0.05). Over 80% of patients demonstrated excellent to good functional outcomes with minimal complications.

Conclusions

Arthroscopic all-inside meniscal repair is a safe, effective, and reliable technique for the management of meniscal tears. It provides excellent short-term functional outcomes with high patient satisfaction and a low complication rate. The procedure supports meniscal preservation and can be expected to yield successful results in the majority of appropriately selected patients.

## Introduction

The knee joint is one of the most complex and frequently injured weight-bearing joints in the human body. Owing to its anatomical configuration and biomechanical demands, the knee is constantly subjected to significant axial loading, shear forces, and rotational stresses during routine activities such as walking, squatting, running, and sports participation [1]. Among the intra-articular structures of the knee, the menisci play a critical role in maintaining joint stability, load transmission, shock absorption, lubrication, and proprioception [2].

The menisci are crescent-shaped fibrocartilaginous structures interposed between the femoral condyles and tibial plateau. They increase the congruity of the tibiofemoral joint and distribute load across a wider surface area, thereby reducing contact stress on the articular cartilage [3]. Biomechanical studies have demonstrated that the menisci transmit approximately 50-70% of the load across the knee joint in extension and up to 85-90% in flexion [4]. Loss of meniscal tissue significantly increases peak contact pressures, accelerating cartilage degeneration and leading to early-onset osteoarthritis [5].

Meniscal injuries are among the most common knee injuries encountered in orthopedic practice, with an annual incidence of approximately 60-70 per 100,000 population [6]. They occur across all age groups and may result from acute traumatic events, particularly in young and active individuals, or from degenerative changes associated with aging and osteoarthritis [7]. Sports activities involving twisting, pivoting, and sudden changes in direction are particularly associated with traumatic meniscal tears [8].

Historically, meniscectomy was considered the standard treatment for symptomatic meniscal tears. However, long-term follow-up studies have clearly demonstrated a strong association between partial or total meniscectomy and the development of early degenerative changes in the knee joint [9]. Fairbank first described characteristic radiological changes, including joint space narrowing and osteophyte formation, following meniscectomy, highlighting the protective role of the meniscus [10]. As a result, contemporary orthopedic practice emphasizes meniscal preservation whenever feasible.

Meniscal repair has emerged as the preferred treatment modality for suitable meniscal tears, particularly in younger patients and in tears located within the vascular zones of the meniscus [11]. Advances in arthroscopic techniques have led to the development of various meniscal repair methods, broadly classified as inside-out, outside-in, and all-inside techniques [12]. Although the inside-out technique is traditionally regarded as the gold standard due to its high healing rates, it is associated with longer operative time, the need for accessory incisions, and potential risk to surrounding neurovascular structures [13].

The all-inside meniscal repair technique was developed to overcome the limitations of conventional methods. This technique allows meniscal repair entirely through arthroscopic portals without the need for additional incisions, thereby minimizing surgical trauma and reducing operative time [14]. Technological advancements in all-inside repair devices have further improved ease of use, fixation strength, and safety, particularly for posterior horn tears, where neurovascular structures are at risk [15].

Despite the increasing popularity of the all-inside technique, concerns have been raised regarding its biomechanical strength, cost, complication rates, and long-term clinical outcomes compared with traditional repair methods [16]. Hence, it is essential to systematically evaluate the functional outcomes, safety, and effectiveness of arthroscopic all-inside meniscal repair using validated clinical outcome measures.

This study aims to assess the functional outcomes of arthroscopic all-inside meniscal repair using standardized scoring systems such as the Lysholm Knee Score, Tegner Activity Scale, International Knee Documentation Committee (IKDC) score, and Visual Analog Scale (VAS), thereby contributing to evidence-based decision-making in meniscal preservation strategies.

## Materials and methods

This prospective observational study was conducted in the Department of Orthopedics at Vydehi Institute of Medical Sciences and Research Centre, a tertiary care teaching hospital, over a period of 18 months from April 2022 to October 2023. The study included 38 patients aged 18-50 years who presented with symptomatic meniscal tears and fulfilled the inclusion criteria. A prospective design was adopted to enable systematic evaluation of functional and clinical recovery following arthroscopic all-inside meniscal repair using validated outcome measures [17,18]. Patients presenting with knee pain, locking, swelling, or instability were evaluated through detailed clinical examination and radiological investigations. Diagnosis and surgical planning were established using MRI, followed by confirmation with diagnostic arthroscopy. Patients with degenerative meniscal tears associated with advanced osteoarthritis (Kellgren-Lawrence grade ≥3), irreparable radial or complex tears, previous knee surgeries, infections, inflammatory arthritis, or neuromuscular disorders were excluded in accordance with established contraindications for meniscal repair [7,10]. Inclusion criteria were selected based on recognized indications for meniscal repair, including repairable tear patterns located in the red-red or red-white zones, with or without associated stable or reconstructed anterior cruciate ligament injury [11-13].

All patients underwent arthroscopic all-inside meniscal repair under spinal or general anesthesia using standard anteromedial and anterolateral portals, with the patient in the supine position. Diagnostic arthroscopy was initially performed to assess tear morphology and stability, followed by preparation of the tear edges using a rasp or shaver to enhance biological healing. Repair was carried out using commercially available all-inside suture devices, with vertical or horizontal mattress sutures placed based on tear configuration and length to achieve stable fixation without overtension. The all-inside technique was preferred due to its advantages of reduced operative time, minimal soft-tissue dissection, and decreased risk of neurovascular injury, particularly in posterior horn repairs [14-16]. Postoperatively, a standardized rehabilitation protocol was implemented, consisting of initial knee immobilization for two weeks, followed by gradual progression from partial to full weight bearing by four to six weeks, early controlled range-of-motion (ROM) exercises with restriction of deep flexion, and delayed return to sports after adequate functional recovery. Early controlled rehabilitation has been shown to promote healing while protecting the repaired meniscus [19].

Functional outcomes were assessed preoperatively and at two weeks, three months, and six months postoperatively using validated scoring systems, including the Lysholm Knee Score, Tegner Activity Scale, IKDC score, and VAS for pain, which are widely accepted tools for evaluating meniscal repair outcomes [20-22]. At each follow-up visit, pain, swelling, knee stability, and ROM were documented. Data were recorded and analyzed using appropriate statistical software, with continuous variables expressed as mean ± SD. Preoperative and postoperative scores were compared using paired t-tests, and a p-value less than 0.05 was considered statistically significant, consistent with standard outcome-based orthopedic research methodology [23]. Ethical approval was obtained from the Institutional Ethics Committee prior to study initiation, and written informed consent was obtained from all participants, ensuring confidentiality and ethical compliance throughout the study.

## Results

A total of 38 patients who underwent arthroscopic all-inside meniscal repair were included in the final analysis. All patients completed the minimum six-month follow-up period and were available for clinical and functional evaluation. The age of the patients ranged from 18 to 50 years, with the majority belonging to the 21- to 30-year-old (16 (42.1%)) age group. This reflects the higher incidence of traumatic meniscal tears among young, active individuals, as reported in previous studies [6,7]. Males (28 (73.7%)) constituted the majority of cases, consistent with the higher participation of males in sports and physically demanding activities. Sports-related injuries (21 (55.3%)) were the most common cause, followed by road traffic accidents (9 (23.7%)) and trivial trauma (8 (21.0%)) (Table 1).

**Table 1:** Demographic and clinical profile of patients

Variable	Category	Number of patients (n = 38)	Percentage (%)
Age (years)	≤20	6	15.8
	21-30	16	42.1
	31-40	10	26.3
	>40	6	15.8
Gender	Male	28	73.7
	Female	10	26.3
Mode of injury	Sports injury	21	55.3
	Road traffic accident	9	23.7
	Trivial fall/twist	8	21
Laterality	Right knee	20	52.6
	Left knee	18	47.4

**Figure 1:**
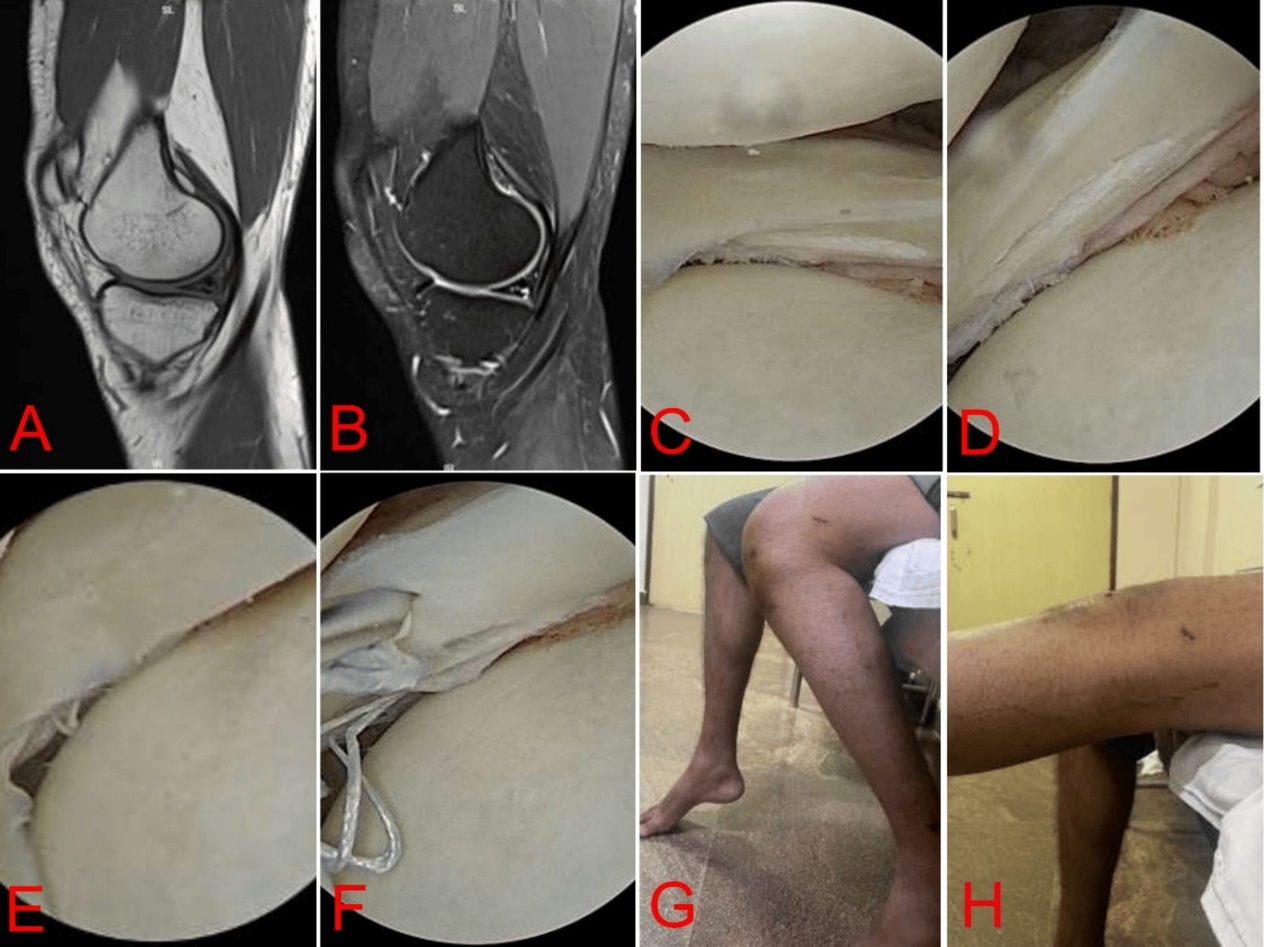
24-year-old male included in the study with an isolated medial meniscal tear at the body and posterior horn (A) Sagittal T1 images showing posterior horn tear of the medial meniscus. (B) Sagittal T2 images showing posterior horn tear of the medial meniscus. (C) Horizontal tear of the body of the medial meniscus. (D) Horizontal tear of the posterior horn of the medial meniscus. (E) Horizontal tear of the body of the medial meniscus repaired using the all-inside technique. (F) Horizontal tear of the posterior horn of the medial meniscus repaired using the all-inside technique. (G) ROM (flexion) up to 120 degrees at the end of six months. (H) Full extension at the end of six months. ROM, range of motion

Functional outcomes were assessed using the Lysholm Knee Score, IKDC score, Tegner Activity Scale, and VAS score preoperatively and at subsequent follow-ups. A progressive and statistically significant improvement was observed in the Lysholm Knee Score at each follow-up interval. The improvement at six months was statistically significant (p < 0.05). There was a marked improvement in knee stability and function as assessed by IKDC scoring. The postoperative improvement was statistically significant (p < 0.05). Patients demonstrated a return toward pre-injury activity levels over time with the Tegner Activity Scale. This functional recovery is comparable with previous all-inside meniscal repair studies [21]. A significant reduction in VAS score was observed (p < 0.05). Pain reduction following meniscal repair has been well documented in the literature [9]. More than 80% of patients achieved good to excellent outcomes (Figure 1, Table 2, Table 3).

**Table 2:** Functional outcome scores at different follow-up intervals The IKDC score was assessed preoperatively and at two weeks, three months, and six months postoperatively. The Tegner Activity Scale and VAS score were assessed preoperatively and at the final six-month follow-up. IKDC, International Knee Documentation Committee; VAS, Visual Analog Scale

Score	Preoperative (mean ± SD)	Two weeks (mean ± SD)	Three months (mean ± SD)	Six months (mean ± SD)	p-Value
Lysholm score	64.4 ± 8.2	84.6 ± 11.02	86.5 ± 11.08	94.05 ± 7.2	<0.05
IKDC score	49.8 ± 9.27	64.03 ± 9.15	71.1 ± 6.7	79.1 ± 4.7	<0.05
Tegner score	3.1 ± 0.9	-	-	5.4 ± 1.1	<0.05
VAS score	2.9 ± 2.7	-	-	1.7 ± 1.08	<0.05

**Table 3:** Functional outcome at six months

Outcome grade	Number of patients	Percentage (%)
Excellent (>90)	24	63.2
Good (84-90)	7	18.4
Fair (65-83)	5	13.2
Poor (<65)	2	5.2

No major complications, such as neurovascular injury, infection, or implant failure, were noted. Minor postoperative pain and transient knee stiffness were observed in a few patients, which resolved with physiotherapy. The low complication rate supports the safety of the all-inside technique [15,16].

## Discussion

Meniscal preservation has become a cornerstone of modern knee surgery due to the well-documented role of the meniscus in load transmission, shock absorption, joint stability, lubrication, and proprioception. The present prospective study evaluated the functional outcomes of arthroscopic all-inside meniscal repair and demonstrated significant improvements in clinical and functional parameters, with a high rate of good to excellent outcomes at short-term follow-up.

In this study, the majority of patients were young adults between 21 and 30 years of age. This age distribution is consistent with epidemiological studies indicating that traumatic meniscal tears are most common in physically active individuals and athletes [6,7]. Younger patients also have superior healing potential due to better vascularity of the peripheral meniscal zones and higher biological reparative capacity [3]. The male predominance observed in this study correlates with previously reported data, where meniscal injuries are more prevalent in males owing to greater participation in sports, manual labor, and high-risk activities [2,8]. Sports-related trauma was the most common mechanism of injury, followed by road traffic accidents and minor twisting injuries. This pattern aligns with studies by Fox et al. and Majewski et al., who identified pivoting and rotational forces during sports activities as major contributors to meniscal tears [6,8]. Acute traumatic tears tend to be longitudinal or vertical in nature and are more amenable to repair, particularly when located in the vascular zones of the meniscus [11].

The study demonstrated a statistically significant improvement in mean Lysholm knee scores from 64.4 ± 8.2 preoperatively to 94.05 ± 7.2 at six months postoperatively. These results are comparable to those reported by Henning et al. and Rockborn and Gillquist, who documented Lysholm scores above 85 following successful meniscal repair [13,24]. High Lysholm scores reflect improvement in pain, stability, and functional capacity, supporting the effectiveness of the all-inside repair technique in restoring knee function.

The IKDC subjective score showed a significant improvement, indicating better patient-perceived knee function and stability. Irrgang et al. emphasized the reliability of the IKDC score in evaluating outcomes following meniscal and ligamentous knee surgery [22]. The findings of this study align closely with contemporary all-inside repair studies, which demonstrate satisfactory short-term functional outcomes [16]. Improvement in Tegner activity scores indicated a gradual return toward pre-injury activity levels. While a complete return to high-level sports may require longer follow-up, the improvement observed in this study is consistent with results reported by Barber and Herbert and Grant et al. [15,16]. The gradual activity progression underscores the importance of postoperative rehabilitation in protecting the repair while optimizing functional recovery.

A significant reduction in pain was noted at the final follow-up, as assessed by the VAS. Pain relief following meniscal repair has been attributed to restoration of meniscal function and stabilization of unstable tear fragments [9]. Similar reductions in postoperative pain have been documented in studies evaluating both inside-out and all-inside meniscal repair techniques [14]. In this study, over 31 (80%) of patients achieved good to excellent outcomes, which is consistent with published success rates ranging from 70% to 90% for meniscal repair procedures [13,15]. This favorable outcome supports meniscal repair as a superior alternative to meniscectomy, particularly in younger and active populations.

No major complications, such as neurovascular injury, implant failure, or deep infection, were observed. The absence of significant complications supports the safety profile of the all-inside technique. Previous studies have highlighted the increased risk of neurovascular injury associated with inside-out repair, particularly in posterior horn tears, which is largely avoided with the all-inside approach [12,15]. Minor complications, such as transient stiffness and postoperative discomfort, resolved with physiotherapy and did not adversely affect functional outcomes.

Several long-term studies have demonstrated a strong association between partial or total meniscectomy and the development of early osteoarthritis [5,9]. Fairbank’s classical description of radiographic changes following meniscectomy emphasizes the importance of meniscal preservation [10]. Compared to meniscectomy, meniscal repair maintains joint biomechanics and delays degenerative changes, making it the preferred treatment option whenever feasible.

Despite encouraging results, the present study has certain limitations. The relatively short follow-up duration limits assessment of long-term healing and degenerative changes. Second-look arthroscopy and postoperative MRI were not routinely performed to confirm biological healing. Additionally, the sample size was relatively small. Larger studies with longer follow-up are required to validate these findings. The results of this study reinforce the role of arthroscopic all-inside meniscal repair as a reliable and effective technique for treating suitable meniscal tears. Proper patient selection, meticulous surgical technique, and structured rehabilitation protocols are critical factors influencing successful outcomes.

## Conclusions

Arthroscopic all-inside meniscal repair is a safe, effective, and reliable surgical technique for managing appropriate meniscal tears, particularly in young and active patients. The procedure results in significant improvement in pain relief, knee stability, and functional outcomes, as evidenced by marked increases in Lysholm, IKDC, and Tegner scores with minimal complications. By preserving meniscal tissue and restoring normal knee biomechanics, all-inside meniscal repair offers a clear advantage over meniscectomy and reinforces its role as a preferred treatment option for suitable meniscal injuries when combined with proper patient selection, meticulous surgical technique, and structured postoperative rehabilitation.

## References

[1] Griffin LY, Agel J, Albohm MJ (2000). Noncontact anterior cruciate ligament injuries: risk factors and prevention strategies. J Am Acad Orthop Surg.

[2] Renström P, Johnson RJ (1990). Anatomy and biomechanics of the menisci. Clin Sports Med.

[3] Makris EA, Hadidi P, Athanasiou KA (2011). The knee meniscus: structure-function, pathophysiology, current repair techniques, and prospects for regeneration. Biomaterials.

[4] Walker PS, Erkman MJ (1975). The role of the menisci in force transmission across the knee. Clin Orthop Relat Res.

[5] Seedhom BB, Dowson D, Wright V (1974). Proceedings: Functions of the menisci. A preliminary study. Ann Rheum Dis.

[6] Fox AJ, Bedi A, Rodeo SA (2012). The basic science of human knee menisci: structure, composition, and function. Sports Health.

[7] Englund M, Guermazi A, Gale D, Hunter DJ, Aliabadi P, Clancy M, Felson DT (2008). Incidental meniscal findings on knee MRI in middle-aged and elderly persons. N Engl J Med.

[8] Majewski M, Susanne H, Klaus S (2006). Epidemiology of athletic knee injuries: a 10-year study. Knee.

[9] Roos H, Adalberth T, Dahlberg L, Lohmander LS (1995). Osteoarthritis of the knee after injury to the anterior cruciate ligament or meniscus: the influence of time and age. Osteoarthritis Cartilage.

[10] Fairbank TJ (1948). Knee joint changes after meniscectomy. J Bone Joint Surg Br.

[11] DeHaven KE (1999). Meniscus repair. Am J Sports Med.

[12] Newman AP, Burks RT (1994). Arthroscopic meniscal repair: “inside-out” technique. Oper Tech Sports Med.

[13] Henning CE, Lynch MA, Clark JR (1987). Vascularity for healing of meniscus repairs. Arthroscopy.

[14] Albrecht-Olsen P, Kristensen G, Burgaard P, Joergensen U, Toerholm C (1999). The arrow versus horizontal suture in arthroscopic meniscus repair. A prospective randomized study with arthroscopic evaluation. Knee Surg Sports Traumatol Arthrosc.

[15] Barber FA, Herbert MA (2000). Meniscal repair devices. Arthroscopy.

[16] Grant JA, Wilde J, Miller BS, Bedi A (2012). Comparison of inside-out and all-inside techniques for the repair of isolated meniscal tears: a systematic review. Am J Sports Med.

[17] Hulley SB, Cummings SR, Browner WS, Grady DG, Newman TB (2013). Designing Clinical Research, 4th Edition. Designing Clinical Research. 4th ed. Philadelphia.

[18] Helms CA (2002). The meniscus: recent advances in MR imaging of the knee. AJR Am J Roentgenol.

[19] Shelbourne KD, Patel DV, Adsit WS, Porter DA (1996). Rehabilitation after meniscal repair. Clin Sports Med.

[20] Lysholm J, Gillquist J (1982). Evaluation of knee ligament surgery results with special emphasis on use of a scoring scale. Am J Sports Med.

[21] Tegner Y, Lysholm J (1985). Rating systems in the evaluation of knee ligament injuries. Clin Orthop Relat Res.

[22] Irrgang JJ, Anderson AF, Boland AL (2001). Development and validation of the International Knee Documentation Committee subjective knee form. Am J Sports Med.

[23] Dawson J, Fitzpatrick R, Murray D, Carr A (1998). Questionnaire on the perceptions of patients about total knee replacement. J Bone Joint Surg Br.

[24] Rockborn P, Messner K (2000). Long-term results of meniscus repair and meniscectomy: a 13-year functional and radiographic follow-up study. Knee Surg Sports Traumatol Arthrosc.

